# Blinatumomab: a bispecific T cell engager (BiTE) antibody against CD19/CD3 for refractory acute lymphoid leukemia

**DOI:** 10.1186/s13045-015-0195-4

**Published:** 2015-09-04

**Authors:** Jingjing Wu, Jiaping Fu, Mingzhi Zhang, Delong Liu

**Affiliations:** Department of Oncology, The first Affiliated Hospital of Zhengzhou University, Zhengzhou, 450052 China; Department of Hematology, Shaoxing People’s Hospital, Shaoxing, Zhejiang Province China; Division of Hematology & Oncology, New York Medical College, Valhalla, NY 10595 USA

## Abstract

Targeted therapy has been the forefront of cancer treatment. Cancer immunotherapy is the most recent focus. In addition, novel immunotherapeutics targeting B cell receptor signaling (e.g., ibrutinib), T cell receptor ( e.g., CART19), and NK cells (e.g., AFM13) are being developed. This review summarized the new development in blinatumomab (MT103/MEDI-538), a first-in-class bispecific T engager (BiTE) antibody against CD19/CD3 in patients with relapsed/refractory precursor B cell acute lymphoid leukemia.

Targeted therapy has been the forefront of cancer treatment [1–7]. Monoclonal antibodies have played a major role in lymphoma therapy for more than a decade [8–11]. Cancer immunotherapy is the most recent focus of clinical development [12–17]. In addition, novel immunotherapeutics targeting B cell receptor signaling (e.g., ibrutinib) [2, 18], T cell receptor (e.g., CART19) [19–22], and NK cells (e.g., AFM13) [23–25] are being developed. This review summarized the clinical development in blinatumomab (MT103/MEDI-538), a first-in-class bispecific T engager (BiTE) antibody against CD19/CD3 in patients with relapsed/refractory precursor B cell acute lymphoid leukemia (ALL).

## Bispecific antibodies and diabody

Bispecific antibodies (bsAb) was initially developed through hybrid-hybridoma, chemical linkage, or renaturation from purified recombinant Fab or Fv fragment from bacterial inclusion bodies [11, 26, 27]. One of the major limitations of these technologies is the difficulty in producing sufficient amount of clinical grade bsAbs. This has made the clinical testing of the bsAbs falling behind.

**Fig. 1:**
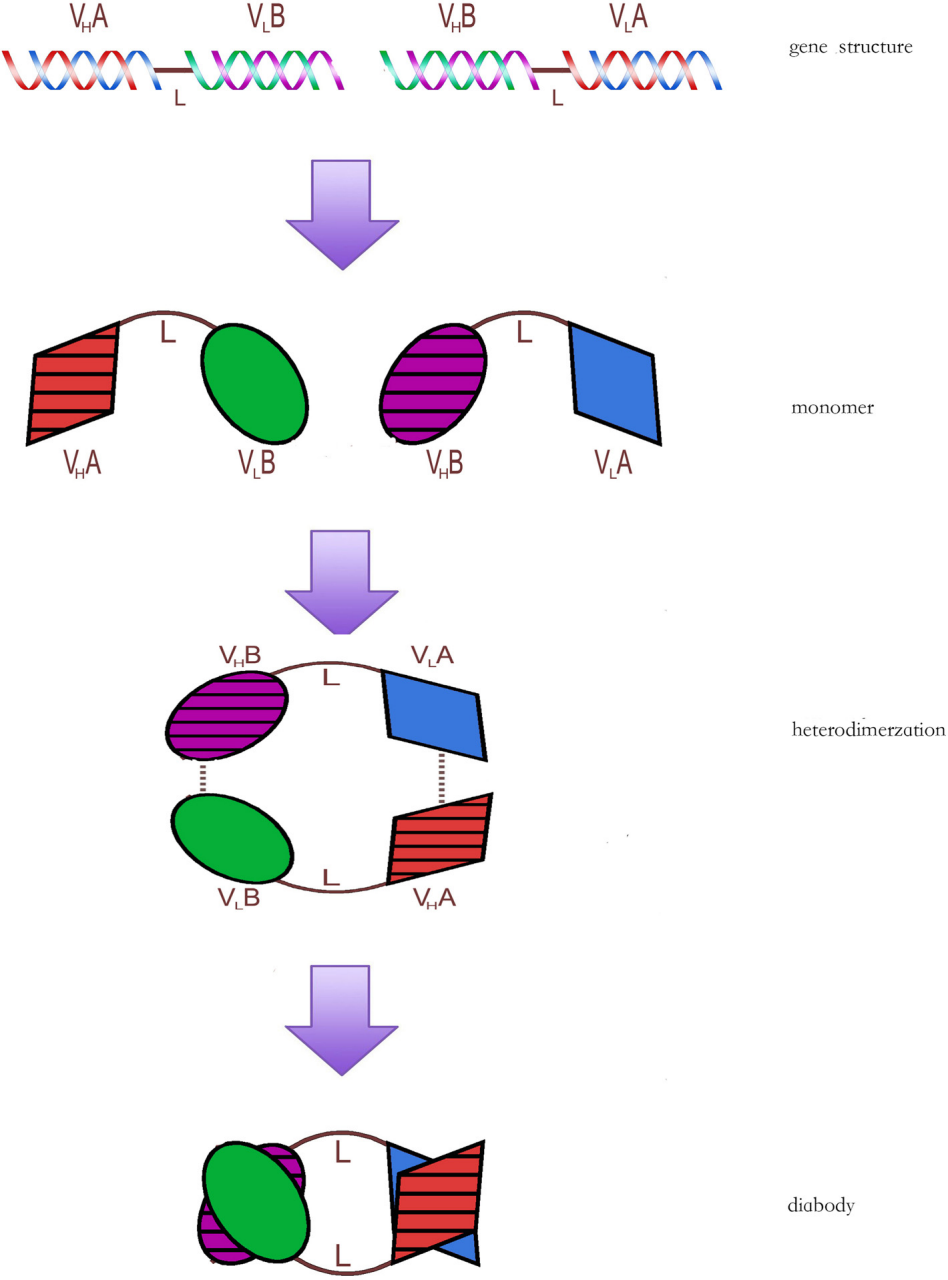
Gene structure and production of bispecific blinatumomab diabody. DNA sequence of the CD19 heavy chain scFv (V_H_A) is connected with the CD3 light chain scFv (V_L_B) by a short linker (L) sequence to form a single gene encoding one peptide, V_H_A-V_L_B. By the same approach, the DNA sequence of the CD19 light chain scFv (V_L_A) is connected with the CD3 heavy chain scFv (V_H_B) by a short linker (L) sequence to form the second gene encoding the other peptide, V_H_B-V_L_A. The two polypeptide chains, V_H_A-V_L_B and V_H_B-V_L_A, can then heterodimerize non-covalently to form a diabody containing bispecific antigen-binding sites to both CD19 and CD3

Through molecular cloning and/or phage expression library, high affinity recombinant single-chain Fv fragment (scFv) has been produced. This led to the development of bivalent bispecific antibody fragments, diabodies [11, 26, 27]. A heavy chain scFv (V_H_) is connected with a light chain scFv (V_L_) by a short amino acid linker to form a single polypeptide. The short linker is too short to allow self association of the two adjacent V_H_ and V_L_ domain. Therefore, by linking the V_H_ and V_L_ of two different antibodies A and B to form two different “cross-over” polypeptide chain V_H_A-V_L_B and V_H_B-V_L_A, a diabody containing both antigen-binding sites through non-covalent association is formed (Fig. 1) [11, 26, 27]. One such functional small bispecific antibody against EpCAM /CD3 was engineered and purified from Chinese hamster ovary (CHO) cells [27]. This antibody was found to be able to redirect T cells to lyse colon cancer cells expression EpCAM antigen. Using this approach, clinical grade bsAbs were produced from CHO cells in large quantity [23, 24, 28].

### Structure and properties of blinatumomab

Combination chemotherapy for relapsed and/or refractory acute lymphoblastic leukemia usually leads to a CR rate in 30–45 % of patients and overall survival of 4·7–8·6 months in first salvage treatment [29–33]. CD19 is a common B cell surface marker [34–38]. Monoclonal antibodies against CD19 have been in active clinical development [39, 40].

**Fig. 2:**
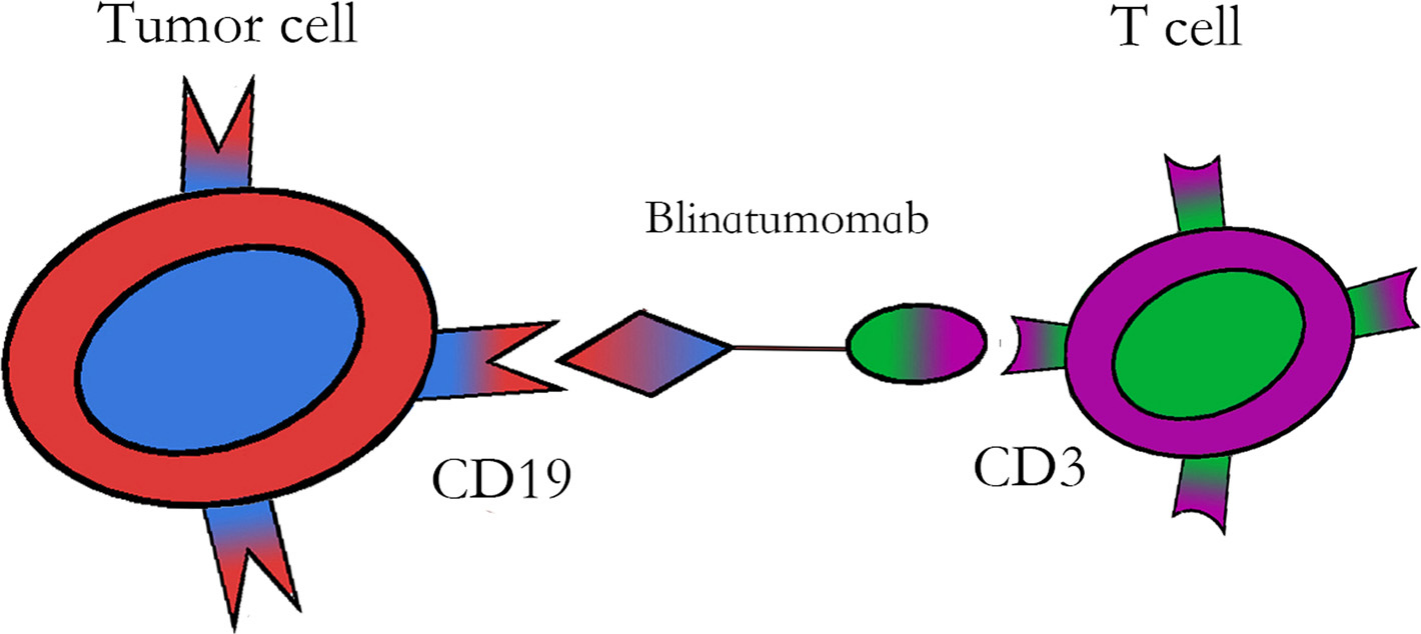
Mechanism of action for blinatumomab as the first-in-class bispecific T cell engager (BiTE). One arm of blinatumomab binds to CD3, the other binds to CD19. This engages the unstimulated T cells which destroy the CD19+ cells

In an attempt to develop novel treatment agent for refractory B cell malignancies, a bsAb against CD19/CD3, MT103/MEDI-538 (blinatumomab), was engineered using the diabody approach [41]. One arm of this antibody binds CD19, while the other arm binds CD3 (Fig. 2). By redirecting unstimulated primary human T cells against CD19-positive lymphoma cells, the bispecific CD19/CD3 antibody fragment showed significant cytotoxic activity at very low concentrations of 10 to 100 pg/mL and at effector-to-target cell ratios as low as 2:1. This single-chain bispecific antibody construct belongs to a new class of antibody fragments, BiTE [42–51]. This bispecific antibody fragment has a molecular weight of 54.1 kDa, approximately one-third of the size of a traditional monoclonal antibody (mAb). As CD19 is an attractive target, CD19 mAb has been widely studied for therapies of lymphoma, leukemia, and autoimmune disorders, such as anti-B4-bR, SAR3419 (huB4-DM4), and BiTE [38–40, 52]. Blinatumomab can potentiate unstimulated T cells and induce direct cytotoxicity against CD19+ cells [42].

Several properties of blinatumomab promoted its development for immunotherapy of lymphoma and leukemia. Because of its single-chain structure, blinatumomab can be produced with a stable purified monomeric formulation in large quantities for clinical use [23, 24, 28, 41]. Blinatumomab has been shown to increase inflammatory cytokine production, specifically IL-2, IFN-γ, TNF-α, IL-4, IL-6, and IL-10 [53]. Importantly, it can bridge malignant B cells directly to CD3-positive T cells, bypassing T cell receptor (TCR) specificity and major histocompatibility complex (MHC) class I molecules [41, 54, 55]. The CD19/CD3 BiTE antibody was shown to induce T-cell-mediated depletion of primary lymphoma cells in 22 out of 25 cases. This effect could be observed at low effector-to-target (E:T) ratios and in the majority of cases without additional activation of autologous T cells by IL-2 [41, 54]. Data from animal models support a high activity of blinatumomab at very low doses against tumor cells in lymphoma and leukemia models [43, 48, 55–57].

### Blinatumomab in clinical development

Blinatumomab is the first-in-class BiTE antibody approved for treatment of refractory ALL [46, 47, 58–64].

Blinatumomab was first reported in a clinical phase I trial in 38 patients with refractory non-Hodgkin lymphoma [58]. Due to its short half-life and mechanism of action, blinatumomab was given as continuous intravenous infusion (CIV) in the study MT103-104 (NCT00274742). The doses ranged from 5 to 60 μg/m^2^/day over a period of 4–8 weeks. The maximum tolerated dose (MTD) of blinatumomab was reported to be 60 μg/m^2^/day. This study first demonstrated the efficacy of blinatumomab in B cell malignancies. Eleven of 38 patients (28.9 %) had measurable response after treatment, including 4 (11 %) CR and 7 (18 %) PR. The most commonly observed adverse events (AEs) were pyrexia, chills, and leucopenia. CNS toxicity and cytokine release syndrome (CRS) were observed [58]. By 2011, 62 patients had been enrolled in this study with an objective response of 18/22 (82 %) and 32-month response duration. Blinatumomab treatment at doses of ≥15 μg/m^2^/day led to depletion of tumor cells in blood, lymph nodes, spleen, and bone marrow [58]. During or after treatment, T cell counts remained stable or increased. Blinatumomab treatment predominantly caused an expansion of effector memory CD8+ and CD4+ T cells with CD45RA/CCR7 phenotype. It was also observed that there was an early disappearance of T cells. This may be due to a transient increase in the adhesiveness of T cells to vessels and/or extravasation. No clinically significant cytokine release syndrome was seen in any patient. There was no autoimmune disorder observed with blinatumomab treatment. Neutralizing antibodies against blinatumomab was not detected in these patients [58].

A single-arm phase II study (MT103-206, NCT01209286) evaluated response to blinatumomab in molecularly relapsed/refractory precursor B cell ALL [61]. In this study, a total of 36 patients were treated. A dose-finding component followed by an extension cohort from 5 (week 1) to 15 μg/m^2^/day on subsequent 3 weeks was included. A 2-week treatment-free interval followed the completion of the 4-week continuous infusion of blinatumomab to allow T cell recovery. CR or CRh (incomplete hematological recovery) was observed in 25 of 36 patients (69 %), and 22 of 25 responders (88 %) achieved a molecular remission. Response was better in patients in first relapse than those in second or greater relapse. Median relapse-free survival (RFS) was 7.6 months, with a 9.8-month median overall survival (OS). With a median follow-up of 405 days, the probability of RFS was 78 %. The most frequent grade 3 and 4 adverse event was lymphopenia, which was reversible. These results are encouraging compared to the median OS of 6 months in relapsed ALL with chemotherapy [61]. Among patients with longer follow-up (median 33 months), 80 % response rate with minimal residual disease (MRD) was reported [65]. Among the 6 Philadelphia chromosome-negative MRD responders, 4 remained in hematologic and molecular remission with no further therapy after blinatumomab. Therefore, blinatumomab can induce long-lasting complete remission in B-lineage ALL patients with persistent or recurrent MRD [65–67].

Subsequently, a large, multicentre, phase II trial (MT103-211, NCT01466179) assessed blinatumomab in 189 adult patients with relapsed or refractory B cell ALL [62, 68]. These patients were negative for Philadelphia chromosome and had primary-refractory disease, early first relapse, or multiple relapses. The trial also enrolled patients who had relapsed within 12 months after allogeneic hematopoietic stem cell transplantation (allo-HSCT). Patients received blinatumomab as continuous intravenous infusion with a flat dose of 9 μg/day at week 1 (cycle 1 only, to reduce cytokine release syndrome) and 28 μg/day for 3 weeks. This was followed by a 2-week treatment-free interval. Therefore, each cycle was 6 weeks, and patients received treatment up to 5 cycles in the study. Premedication with dexamethasone (20 mg) within 1 h of treatment initiation in each cycle and before the dose step-up in cycle 1 was given to minimize infusion reactions to blinatumomab. However, high-dose (≥24 mg) dexamethasone was only allowed for 7 days or less. The infusion was allowed to be interrupted for AEs grade 3 or higher and resumed after reduction of AEs to grade I or complete resolution. The clinical response in the first 2 cycles was 33 % CR (63 of 189 patients) and 10 % CRh (18 of 189 patients), with 40 % (32 of 81 patients in CR/CRh) went on to receive allo-HSCT. Median relapse-free survival was 5∙9 months for those patients who achieved CR/CRh, with a median OS of 6∙1 month for all 189 patients [68]. Among the 73 responders with available data on MRD, 80 % of patients achieved MRD negativity [69]. The relative odds ratio by Mantel-Byar analysis for survival benefit of achieving remission was 0.13 (*p* < 0∙0001). Responses in patients older than 65 years and in those who received previous allo-HSCT were noted. These two patient groups for whom treatment options are very limited due to substantial toxicity associated with currently available polychemotherapies. Common AEs associated with blinatumomab such as febrile neutropenia, neutropenia, and anemia were consistent with those previously reported. CRS in grade 3 occurred in 3 (2 %) patients [68, 70]. Neurologic events, including tremors, seizure, and mental status change, were seen in 98 (52 %) patients, mostly in grade 1 or 2, with 20 (11 %) in grade 3 and 4 (2 %) in grade 4. In these patients with high-risk features, 40 % of them went on to allo-HSCT (17 % for those with prior transplantation, 52 % with no prior transplantation). There were patients who had response to blinatumomab but did not receive allo-HSCT because they were ≥65 or had prior HSCT that precluded them from allo-HSCT [71].

In a separate confirmatory trial, BLAST trial, 116 adult patients (median age 45, range 18–76) with MRD+ pre-B ALL were treated with continuous IV infusion of blinatumomab at 15 μg/m^2^/day in a similar schedule (4-week treatment, 2-week rest) as described above [72]. The MRD negative response rate was 78 % (95 % CI, 69–85 %) after 1 cycle of treatment. The results appeared to be consistent with those from previous studies [65, 68].

Blinatumomab was also studied in pediatric patients with relapsed/refractory pre-B ALL in a phase I/II clinical trial [73]. Patients received blinatumomab for 4 weeks by continuous IV infusion followed by a 2-week treatment-free period (for up to 5 cycles). Escalating dosing levels of 5, 15, and 30 μg/m^2^/day and stepwise dosing of 5–15 or 15–30 μg/m^2^/day were evaluated. In the phase I portion, 41 patients were treated. A total of 13 (32 %) patients achieved CR with 10 (77 %) achieving MRD negativity. Of these 13 patients, 9 (69 %) went on to have HSCT. The adverse events as well as pharmacokinetic data, including steady-state concentration, clearance, and half-life were similar to those from adult patients with relapsed/refractory BCP-ALL who received body surface area-based dosing. The MTD was 15 μg/m^2^/day in the pediatric patients. Stepwise dosing was useful in reducing CRS. The 5 (week 1) to 15 μg/m^2^/day (week 2 to 4) step-wise dosing was therefore used for phase II portion of the study. At the time of last update, 39 patients were treated at this dose (median age 9, range 2–18) [74]. Blinatumomab showed promising antileukemia activity in this group of high-risk pediatric relapsed/refractory B cell precursor ALL patients. Among patients who had remission after the first 2 cycles of blinatumomab single-agent therapy, half went on to receive allo-HSCT. Therefore, blinatumomab may create a window for allo-HSCT for those patients who are resistant to salvage chemotherapy [73–75].

### Conclusion and future directions

Blinatumomab represents the first-in-class BiTE antibody in clinical use and provides a novel therapeutic option for patients with relapsed/refractory B cell ALL [43, 58, 64, 67, 76]. The pharmacodynamics and immunophenotype data are still being collected [67]. One obvious disadvantage of this BiTE antibody is the requirement for continuous IV infusion because of the small molecular weight and rapid clearance from circulation. Newer tetravalent bispecific antibodies, AFM11 and AFM13, can be given as weekly or twice weekly [23, 24, 28]. T cells with CD19/CD3 chimeric antigen receptors (CAR-T) have been shown to induce high remission rate (90 % CR in refractory ALL), and can expand 1000 times in vivo [77–80]. The rate of CRS associated with CAR-T therapy (27 % severe) was much higher than that of blinatumomab (2 %). CAR-T was shown to penetrate blood-brain barrier [78]. It is not known whether blinatumomab has similar property. It remains unclear what is the optimal treatment duration and schedule of blinatumomab for patients who cannot receive allo-HSCT. The role of consolidation or maintenance for blinatumomab also remains an area of investigation. Incorporation of blinatumomab in the first-line treatment setting is in active clinical trials (NCT02143414, phase II and NCT02003222, phase III). BiTE antibodies against other antigens (e.g., CD33, CD 79b) are under active clinical studies for myeloid leukemia and lymphoma [81, 82]. Since blinatumomab was shown to activate effector T cells [52, 58], it would be interesting to study the potential of using blinatumomab for effector T cell expansion for cancer immunotherapy.

## References

[1] Akinleye A, Avvaru P, Furqan M, Song Y, Liu D (2013). Phosphatidylinositol 3-kinase (PI3K) inhibitors as cancer therapeutics. J Hematol Oncol.

[2] Akinleye A, Chen Y, Mukhi N, Song Y, Liu D (2013). Ibrutinib and novel BTK inhibitors in clinical development. J Hematol Oncol.

[3] Akinleye A, Furqan M, Mukhi N, Ravella P, Liu D (2013). MEK and the inhibitors: from bench to bedside. J Hematol Oncol.

[4] Furqan M, Akinleye A, Mukhi N, Mittal V, Chen Y, Liu D (2013). STAT inhibitors for cancer therapy. J Hematol Oncol.

[5] Furqan M, Mukhi N, Lee B, Liu D (2013). Dysregulation of JAK-STAT pathway in hematological malignancies and JAK inhibitors for clinical application. Biomarker Res.

[6] Huang T, Karsy M, Zhuge J, Zhong M, Liu D (2013). B-Raf and the inhibitors: from bench to bedside. J Hematol Oncol.

[7] Saha M, Qiu L, Chang H (2013). Targeting p53 by small molecules in hematological malignancies. J Hematol Oncol.

[8] Goede V, Fischer K, Busch R, Engelke A, Eichhorst B, Wendtner CM (2014). Obinutuzumab plus chlorambucil in patients with CLL and coexisting conditions. N Engl J Med.

[9] Younes A, Bartlett NL, Leonard JP, Kennedy DA, Lynch CM, Sievers EL (2010). Brentuximab vedotin (SGN-35) for relapsed CD30-positive lymphomas. N Engl J Med.

[10] Mellor J, Brown M, Irving H, Zalcberg J, Dobrovic A (2013). A critical review of the role of Fc gamma receptor polymorphisms in the response to monoclonal antibodies in cancer. J Hematol Oncol.

[11] Suresh T, Lee L, Joshi J, Barta S (2014). New antibody approaches to lymphoma therapy. J Hematol Oncol.

[12] Ansell SM, Lesokhin AM, Borrello I, Halwani A, Scott EC, Gutierrez M (2015). PD-1 blockade with nivolumab in relapsed or refractory Hodgkin’s lymphoma. N Engl J Med.

[13] Garon EB, Rizvi NA, Hui R, Leighl N, Balmanoukian AS, Eder JP (2015). Pembrolizumab for the treatment of non-small-cell lung cancer. N Engl J Med.

[14] Postow MA, Callahan MK, Wolchok JD (2015). Immune checkpoint blockade in cancer therapy. J Clin Oncol.

[15] Postow MA, Chesney J, Pavlick AC, Robert C, Grossmann K, McDermott D (2015). Nivolumab and ipilimumab versus ipilimumab in untreated melanoma. N Engl J Med.

[16] Robert C, Long GV, Brady B, Dutriaux C, Maio M, Mortier L (2015). Nivolumab in previously untreated melanoma without BRAF mutation. N Engl J Med.

[17] Robert C, Schachter J, Long GV, Arance A, Grob JJ, Mortier L (2015). Pembrolizumab versus ipilimumab in advanced melanoma. N Engl J Med.

[18] Novero A, Ravella P, Chen Y, Dous G, Liu D (2014). Ibrutinib for B cell malignancies. Exp Hematol Oncol.

[19] Han E, Li X-l, Wang C-r, Li T-f, Han S-y (2013). Chimeric antigen receptor-engineered T cells for cancer immunotherapy: progress and challenges. J Hematol Oncol.

[20] Haso W, Lee DW, Shah NN, Stetler-Stevenson M, Yuan CM, Pastan IH (2012). Anti-CD22-chimeric antigen receptors targeting B-cell precursor acute lymphoblastic leukemia. Blood.

[21] Ruella M, Barrett D, Kenderian SS, Shestova O, Hofmann TJ, Scholler J (2014). Novel chimeric antigen receptor T Cells for the treatment of CD19-negative relapses occurring after CD19-targeted immunotherapies. Blood.

[22] Chen Y, Liu D (2014). Chimeric antigen receptor (CAR)-directed adoptive immunotherapy: a new era in targeted cancer therapy. Stem Cell Investigation.

[23] Reusch U, Burkhardt C, Fucek I, Le Gall F, Le Gall M, Hoffmann K (2014). A novel tetravalent bispecific TandAb (CD30/CD16A) efficiently recruits NK cells for the lysis of CD30+ tumor cells. mAbs.

[24] Rothe A, Sasse S, Topp MS, Eichenauer DA, Hummel H, Reiners KS (2015). A phase 1 study of the bispecific anti-CD30/CD16A antibody construct AFM13 in patients with relapsed or refractory Hodgkin lymphoma. Blood.

[25] Wu J, Fu J, Zhang M, Liu D (2015). AFM13: a first-in-class tetravalent bispecific anti-CD30/CD16A antibody for NK cell-mediated immunotherapy. J Hematol Oncol.

[26] Holliger P, Prospero T, Winter G (1993). “Diabodies”: small bivalent and bispecific antibody fragments. Proc Natl Acad Sci U S A.

[27] Mack M, Riethmuller G, Kufer P (1995). A small bispecific antibody construct expressed as a functional single-chain molecule with high tumor cell cytotoxicity. Proc Natl Acad Sci U S A.

[28] Reusch U, Duell J, Ellwanger K, Herbrecht C, Knackmuss SH, Fucek I (2015). A tetravalent bispecific TandAb (CD19/CD3), AFM11, efficiently recruits T cells for the potent lysis of CD19(+) tumor cells. mAbs.

[29] Forman SJ, Rowe JM (2012). The myth of the second remission of acute leukemia in the adult. Blood.

[30] Goekbuget N, Hoelzer D (2009). Treatment of adult acute lymphoblastic leukemia. Semin Hematol.

[31] Rowe JM, Goldstone AH (2007). How I treat acute lymphocytic leukemia in adults. Blood.

[32] Mathisen MS, Jabbour E, Kantarjian HM (2012). Treatment of adult acute lymphoblastic leukemia (ALL) with a focus on emerging investigational and targeted therapies. Oncology (Williston Park).

[33] Zhao Y, Huang H, Wei G (2013). Novel agents and biomarkers for acute lymphoid leukemia. J Hematol Oncol.

[34] Carter RH, Myers R (2008). Germinal center structure and function: lessons from CD19. Semin Immunol.

[35] Carter RH, Wang Y, Brooks S (2002). Role of CD19 signal transduction in B cell biology. Immunol Res.

[36] Chung EY, Psathas JN, Yu D, Li Y, Weiss MJ, Thomas-Tikhonenko A (2012). CD19 is a major B cell receptor-independent activator of MYC-driven B-lymphomagenesis. J Clin Invest.

[37] Del Nagro CJ, Otero DC, Anzelon AN, Omori SA, Kolla RV, Rickert RC (2005). CD19 function in central and peripheral B-cell development. Immunol Res.

[38] Wang K, Wei G, Liu D (2012). CD19: a biomarker for B cell development, lymphoma diagnosis and therapy. Exp Hematol Oncol.

[39] Blanc V, Bousseau A, Caron A, Carrez C, Lutz RJ, Lambert JM (2011). SAR3419: an anti-CD19-maytansinoid Immunoconjugate for the treatment of B-cell malignancies. Clin Cancer Res.

[40] Breton C, Nahimana A, Aubry D, Macoin J, Moretti P, Bertschinger M (2014). A novel anti-CD19 monoclonal antibody (GBR 401) with high killing activity against B cell malignancies. J Hematol Oncol.

[41] Loffler A, Kufer P, Lutterbuse R, Zettl F, Daniel PT, Schwenkenbecher JM (2000). A recombinant bispecific single-chain antibody, CD19 x CD3, induces rapid and high lymphoma-directed cytotoxicity by unstimulated T lymphocytes. Blood.

[42] Brischwein K, Parr L, Pflanz S, Volkland J, Lumsden J, Klinger M (2007). Strictly target cell-dependent activation of T cells by bispecific single-chain antibody constructs of the BiTE class. J Immunother.

[43] Buie LW, Pecoraro JJ, Horvat TZ, Daley RJ (2015). Blinatumomab: a first-in-class bispecific T-Cell engager for precursor B-cell acute lymphoblastic leukemia. Ann Pharmacother.

[44] d’Argouges S, Wissing S, Brandl C, Prang N, Lutterbuese R, Kozhich A (2009). Combination of rituximab with blinatumomab (MT103/MEDI-538), a T cell-engaging CD19-/CD3-bispecific antibody, for highly efficient lysis of human B lymphoma cells. Leuk Res.

[45] Zimmerman Z, Maniar T, Nagorsen D (2014). Unleashing the clinical power of T cells: CD19/CD3 bi-specific T cell engager (BiTE(R)) antibody construct blinatumomab as a potential therapy. Int Immunol.

[46] Nagorsen D, Kufer P, Baeuerle PA, Bargou R (2012). Blinatumomab: a historical perspective. Pharmacol Ther.

[47] Oak E, Bartlett NL (2015). Blinatumomab for the treatment of B-cell lymphoma. Expert Opin Investig Drugs.

[48] Rogala B, Freyer CW, Ontiveros EP, Griffiths EA, Wang ES, Wetzler M (2015). Blinatumomab: enlisting serial killer T-cells in the war against hematologic malignancies. Expert Opin Biol Ther.

[49] Bassan R (2012). Toward victory in adult ALL: blinatumomab joins in. Blood.

[50] Nagorsen D, Baeuerle PA (2011). Immunomodulatory therapy of cancer with T cell-engaging BiTE antibody blinatumomab. Exp Cell Res.

[51] Nagorsen D, Bargou R, Ruttinger D, Kufer P, Baeuerle PA, Zugmaier G (2009). Immunotherapy of lymphoma and leukemia with T-cell engaging BiTE antibody blinatumomab. Leuk Lymphoma.

[52] Golay J, D’Amico A, Borleri G, Bonzi M, Valgardsdottir R, Alzani R (2014). A novel method using blinatumomab for efficient, clinical-grade expansion of polyclonal T cells for adoptive immunotherapy. J Immunol.

[53] Brandl C, Haas C, d’Argouges S, Fisch T, Kufer P, Brischwein K (2007). The effect of dexamethasone on polyclonal T cell activation and redirected target cell lysis as induced by a CD19/CD3-bispecific single-chain antibody construct. Cancer Immunol Immunother.

[54] Loffler A, Gruen M, Wuchter C, Schriever F, Kufer P, Dreier T (2003). Efficient elimination of chronic lymphocytic leukaemia B cells by autologous T cells with a bispecific anti-CD19/anti-CD3 single-chain antibody construct. Leukemia.

[55] Leone P, Shin EC, Perosa F, Vacca A, Dammacco F, Racanelli V (2013). MHC class I antigen processing and presenting machinery: organization, function, and defects in tumor cells. J Natl Cancer Inst.

[56] Frankel SR, Baeuerle PA (2013). Targeting T cells to tumor cells using bispecific antibodies. Curr Opin Chem Biol.

[57] Klinger M, Brandl C, Zugmaier G, Hijazi Y, Bargou RC, Topp MS (2012). Immunopharmacologic response of patients with B-lineage acute lymphoblastic leukemia to continuous infusion of T cell-engaging CD19/CD3-bispecific BiTE antibody blinatumomab. Blood.

[58] Bargou R, Leo E, Zugmaier G, Klinger M, Goebeler M, Knop S (2008). Tumor regression in cancer patients by very low doses of a T cell-engaging antibody. Science.

[59] Hoffman LM, Gore L (2014). Blinatumomab, a bi-Specific anti-CD19/CD3 BiTE((R)) antibody for the treatment of aAcute lymphoblastic leukemia: perspectives and current pediatric applications. Front Oncol.

[60] Topp MS, Kufer P, Gokbuget N, Goebeler M, Klinger M, Neumann S (2011). Targeted therapy with the T-cell-engaging antibody blinatumomab of chemotherapy-refractory minimal residual disease in B-lineage acute lymphoblastic leukemia patients results in high response rate and prolonged leukemia-free survival. J Clin Oncol.

[61] Topp MS, Gokbuget N, Zugmaier G, Klappers P, Stelljes M, Neumann S (2014). Phase II trial of the anti-CD19 bispecific T cell-engager blinatumomab shows hematologic and molecular remissions in patients with relapsed or refractory B-precursor acute lymphoblastic leukemia. J Clin Oncol.

[62] Topp MS, Gockbuget N, Stein AS (2015). Correction to Lancet Oncol 2015; 16: 60, 61. Safety and activity of blinatumomab for adult patients with relapsed or refractory B-precursor acute lymphoblastic leukaemia: a multi-centre, single-arm, phase 2 study. Lancet Oncol.

[63] Advani AS (2012). Blinatumomab: a novel agent to treat minimal residual disease in patients with acute lymphoblastic leukemia. Clin Adv Hematol Oncol.

[64] Traynor K (2015). Blinatumomab approved for rare leukemia. Am J Health Syst Pharm.

[65] Topp MS, Gokbuget N, Zugmaier G, Degenhard E, Goebeler ME, Klinger M (2012). Long-term follow-up of hematologic relapse-free survival in a phase 2 study of blinatumomab in patients with MRD in B-lineage ALL. Blood.

[66] Zugmaier G, Goekbuget N, Viardot A, Stelljes M, Neumann S, Horst HA (2014). Long-term survival in adult patients with relapsed/refractory B-precursor acute lymphoblastic leukemia (ALL) who achieved minimal residual disease (MRD) response following anti-CD19 BiTE® blinatumomab. Blood.

[67] Zugmaier G, Topp MS, Alekar S, Viardot A, Horst HA, Neumann S (2014). Long-term follow-up of serum immunoglobulin levels in blinatumomab-treated patients with minimal residual disease-positive B-precursor acute lymphoblastic leukemia. Blood Cancer J.

[68] Topp MS, Gokbuget N, Stein AS, Zugmaier G, O’Brien S, Bargou RC (2014). Safety and activity of blinatumomab for adult patients with relapsed or refractory B-precursor acute lymphoblastic leukaemia: a multicentre, single-arm, phase 2 study. Lancet Oncol.

[69] Goekbuget N, Kantarjian H, Brüggemann M, Stein A, Bargou RC, Dombret H (2014). An evaluation of molecular response in a phase 2 open-label, multicenter confirmatory study in patients (pts) with relapsed/refractory B-precursor acute lymphoblastic leukemia (r/r ALL) receiving treatment with the BiTE® antibody construct blinatumomab. Blood.

[70] Teachey DT, Rheingold SR, Maude SL, Zugmaier G, Barrett DM, Seif AE (2013). Cytokine release syndrome after blinatumomab treatment related to abnormal macrophage activation and ameliorated with cytokine-directed therapy. Blood.

[71] Stein A, Topp MS, Goekbuget N, Bargou RC, Dombret H, Fielding AK (2014). Allogeneic hematopoietic stem cell transplantation following anti-CD19 BiTE® blinatumomab in adult patients with relapsed/refractory B-precursor acute lymphoblastic leukemia (ALL). Blood.

[72] Goekbuget N, Dombret H, Bonifacio M, Reichle A, Graux C, Havelange V (2014). BLAST: a confirmatory, single-arm, phase 2 study of blinatumomab, a bispecific T-cell engager (BiTE®) antibody construct, in patients with minimal residual disease B-precursor acute lymphoblastic leukemia (ALL). Blood.

[73] von Stackelberg A, Locatelli F, Zugmaier G, Handgretinger R, Trippett TM, Rizzari C (2014). Phase 1/2 study in pediatric patients with relapsed/refractory B-cell precursor acute lymphoblastic leukemia (BCP-ALL) receiving blinatumomab treatment. Blood.

[74] Gore L, Locatelli F, Zugmaier G, Zwaan CM, Bhojwani D, Handgretinger R (2014). Initial results from a phase 2 study of blinatumomab in pediatric patients with relapsed/refractory B-cell precursor acute lymphoblastic leukemia. Blood.

[75] Handgretinger R, Zugmaier G, Henze G, Kreyenberg H, Lang P, von Stackelberg A (2010). Complete remission after blinatumomab-induced donor T-cell activation in three pediatric patients with post-transplant relapsed acute lymphoblastic leukemia. Leukemia.

[76] Wong R, Pepper C, Brennan P, Nagorsen D, Man S, Fegan C (2013). Blinatumomab induces autologous T-cell killing of chronic lymphocytic leukemia cells. Haematologica.

[77] Kalos M, Levine BL, Porter DL, Katz S, Grupp SA, Bagg A (2011). T cells with chimeric antigen receptors have potent antitumor effects and can establish memory in patients with advanced leukemia. Sci Translational Med.

[78] Porter DL, Levine BL, Kalos M, Bagg A, June CH (2011). Chimeric antigen receptor-modified T cells in chronic lymphoid leukemia. N Engl J Med.

[79] Sadelain M, Brentjens R, Riviere I (2013). The basic principles of chimeric antigen receptor design. Cancer Discovery.

[80] Brentjens RJ, Davila ML, Riviere I, Park J, Wang X, Cowell LG (2013). CD19-targeted T cells rapidly induce molecular remissions in adults with chemotherapy-refractory acute lymphoblastic leukemia. Sci Translational Med.

[81] Krupka C, Brauneck F, Lichtenegger FS, Kufer P, Kischel R, Zugmaier G (2014). Hydroxyurea is most suitable for cytoreduction of AML prior to CD33/CD3 bispecific BiTE® antibody (AMG 330) therapy: uncompromised T-cell proliferation ex-vivo and CD33 upregulation on AML cells. Blood.

[82] Sun LL, Chen X, Chen Y, Dennis MS, Ellerman D, Johnson C (2014). Pre-clinical characterization of T cell-dependent bispecific antibody anti-CD79b/CD3 as a potential therapy for B cell malignancies. Blood.

